# Xbox Kinect Sports Effects on Cognition Status and Physical Performance in Physically Inactive Older Females: A Randomized Controlled Trial

**DOI:** 10.3390/jcm14072165

**Published:** 2025-03-22

**Authors:** Edgar Vásquez-Carrasco, Celia Sánchez Gómez, Pablo Valdés-Badilla, Jordan Hernandez-Martinez, Francisca Villagrán-Silva, Cristian Sandoval, Pedro Moruno Miralles

**Affiliations:** 1 School of Occupational Therapy, Faculty of Psychology, University of Talca, Talca 3465548, Chile; edgar.vasquez@utalca.cl; 2 Centro de Investigación en Ciencias Cognitivas, Faculty of Psychology, Universidad de Talca, Talca 3465548, Chile; 3 Department of Developmental and Educational Psychology, University of Salamanca, 37008 Salamanca, Spain; 4 Institute of Biomedical Research of Salamanca (IBSAL), 37008 Salamanca, Spain; 5 Department of Physical Activity Sciences, Faculty of Education Sciences, Universidad Católica del Maule, Talca 3530000, Chile; 6 Sports Coach Career, School of Education, Universidad Viña del Mar, Viña del Mar 2520000, Chile; 7 Department of Physical Activity Sciences, Universidad de Los Lagos, Osorno 5290000, Chile; jordan.hernandez@ulagos.cl; 8 G-IDyAF Research Group, Department of Physical Activity Sciences, Universidad de Los Lagos, Osorno 5290000, Chile; 9 Programa de Investigación en Deporte, Sociedad y Buen Vivir, Universidad de los Lagos, Osorno 5290000, Chile; 10 Programa de Doctorado en Ciencias Morfológicas, Facultad de Medicina, Universidad de La Frontera, Temuco 4811230, Chile; f.villagran04@ufromail.cl; 11 Escuela de Tecnología Médica, Facultad de Salud, Universidad Santo Tomás, Osorno 5310431, Chile; 12 Departamento de Medicina Interna, Facultad de Medicina, Universidad de La Frontera, Temuco 4811230, Chile; 13 Núcleo Científico y Tecnológico en Biorecursos (BIOREN), Universidad de La Frontera, Temuco 4811230, Chile; 14 Department of Nursing, Physiotherapy and Occupational Therapy, University of Castilla-La Mancha, 45600 Toledo, Spain; pedro.moruno@uclm.es

**Keywords:** aging, cognition, exergaming, physical functional performance, virtual reality exercise

## Abstract

**Background/Objectives:** This study aimed to compare the effects of Xbox Kinect Sports (XKS) relative to an inactive control group (CG) on cognitive status and physical performance in physically inactive older females. **Methods:** A randomized controlled trial study was conducted with the following groups: XKS (n = 15) and CG (n = 15), considering three weekly sessions of 60 min for 24 weeks. A two-way mixed analysis of variance with repeated measures was performed. **Results**: A two-way mixed ANOVA revealed significant time × group interactions for the Mini-Mental State Examination (MMSE) (F_(2,18)_ = 14.8, *p* = 0.000, ηp^2^ = 0.292, large effect), Timed Up-and-Go (TUG) (F_(2,18)_ = 19.5, *p* = 0.000, ηp^2^ = 0.351, large effect), and Falls Efficacy Scale-International (FES-I) (F_(2,18)_ = 6.55, *p* = 0.015, ηp^2^ = 0.154, large effect). No significant interactions were observed for the Berg Balance Scale (BBS) (F_(2,18)_ = 0.998, *p* = 0.324, ηp^2^ = 0.027, small effect), Maximal Isometric Handgrip Strength (MIHS) (dominant: F_(2,18)_ = 0.163, *p* = 0.688, ηp^2^ = 0.005; non-dominant: F_(2,18)_ = 0.012, *p* = 0.912, ηp^2^ = 0.000, small effects), or Maximal Isometric Pinch Strength (MIPS) (dominant: F_(2,18)_ = 0.099, *p* = 0.756, ηp^2^ = 0.003; non-dominant: F_(2,18)_ = 0.233, *p* = 0.632, ηp^2^ = 0.006, small effects). **Conclusions:** XKS significantly improves cognitive status measured by the MMSE and physical performance through reduced time in TUG and fear of falling through the FES-I in physically inactive older females.

## 1. Introduction

The aging of the world’s population has generated a significant increase in functional dependence and cognitive impairment, which compromises the independence of older people and their social participation [1]. Approximately, 40% of people over 65 years of age have problems performing basic activities of daily living, such as dressing, bathing or walking without assistance, and their instrumental activities of daily living [2]. To promote independence and autonomy in older people, incorporating cognitive stimulation and physical activities into their daily routines and lifestyles is essential [3]. Findings indicate that conventional interventions, such as muscle strength and endurance exercises and the combination of physical activity and cognitive stimulation, significantly improve memory, attention and executive functions, this physical activity and cognitive stimulation a key role in promoting healthy aging and preventing cognitive decline and dependence in activities of daily living [4]. Strategies that combine physical activity and cognitive stimulation not only improve global cognition but also gait performance, suggesting a positive impact on delaying the aging process and maintaining quality of life in old age [5]. Therefore, physical activity and cognitive stimulation improves cognitive status and physical performance in older people, leading to greater independence in activities of daily living [6].

Physical activity and exercise are essential for counteracting physical and cognitive decline in aging, which is why exergames, a therapy combining physical activity and cognitive stimulation, have emerged as an effective option within non-traditional interventions [7]. Exergaming may mitigate cognitive decline associated with aging in healthy older people, showing positive effects on executive functions and neurophysiological correlates [8]. Devices that offer an innovative form of intervention not only generate enjoyment and promote health but also reduce cognitive risks, falls, and disability these devices play a crucial role in enhancing physical and cognitive well-being, providing a promising approach to improving the quality of life for older people [9]. Yen and Chiu [10] suggest that exergames may provide potential positive influences on cognition, memory, depression, and increasing independence in older people. Another study suggests that exergame-based dual-task training improved both executive function dual-task cognitive performance and dual-task motor performance, improving community walking ability [11]. One of the exergames with the most evidence is that training with Xbox Kinect Sports (XKS), released in 2009, has also been used for therapeutic purposes; this console is controlled through the player’s movements, eliminating the need for a controller [12]. It has been shown to significantly reduce body fat percentage and time on timed up-and-go (TUG) test, while significantly improving performance on 30-s chair stand test, sit-and-reach test, and the 2 min step in physically inactive older females [13]. Another study found that the developed XKS was effective in improving gait speed, lower body strength, and postural balance in older people [14]. Exergames, such as XKS, being interactive and novel, provide additional benefits to those already mentioned, such as greater adherence and enjoyment during training [15].

The frequency of sessions and the time in weeks seem to be important to have more effective results. For example, studies lasting 6 to 8 weeks have shown moderate significant results [16,17]. On the other hand, studies lasting between 12 and 24 weeks obtained significant results in the variables of cognitive status and physical performance compared to active control group (CG) [18]. The presence of an inactive CG allows the observed effects to be attributed exclusively to the intervention, eliminating the possibility that other external factors explain the results [19,20]. This study aimed to compare the effects of XKS versus an inactive control group on cognitive status (MMSE) and physical performance (TUG, BBS, FES-I, MIHS, and MIPS) in physically inactive older females. Based on previous studies [21,22], we hypothesize that XKS produces significantly better results on MMSE, TUG, BBS, FES-I, MIHS, and MIPS compared to CG.

## 2. Materials and Methods

### 2.1. Study Design

Randomization was carried out using a sealed-envelope lottery within a single-blinded design (evaluators), structured as a two-arm randomized controlled trial (RCT): XKS group and CG. The randomization process was facilitated by the Randomizer website (https://www.randomizer.org; accessed on 1 July 2023), following the methodology outlined in the CONSORT guidelines [23]. This study was registered in the US Clinical Trials Registry and Outcomes System under the code NCT05275140 (https://clinicaltrials.gov/search?cond=NCT05275140; accessed on 21 December 2024), with its initial publication dated 11 July 2024.

### 2.2. Participants

The intervention initially included 42 healthy older females recruited from health centers. Participants underwent interventions conducted 3 times per week over 24 weeks, totaling 72 sessions. These sessions were performed thrice a week (Monday, Wednesday, and Friday) for 60 min each. Participants in the XKS group engaged in 60 min sports video game sessions, while those in the CG maintained their usual daily routines. All assessments took place in the afternoon, between 15:00 and 17:00, at a consistent location (a community center in Osorno, Chile). No musculoskeletal or cardiorespiratory injuries were reported among the older females during the intervention, nor was pain experienced before assessments or during training. Six participants withdrew: 3 due to lack of motivation, 1 because of health issues, 1 due to vision impairment, and 1 for failing to complete all re-assessments.

### 2.3. Selection Criteria

The inclusion criteria were (i) females aged between 60 and 65 years; (ii) no visual difficulties or vestibular disorders that would hinder the performance of the games on the screen; (iii) independent, with at least 43 points on the Preventive Medicine Examination for Older People from the Chilean Ministry of Health [24]; (iv) able to meet the attendance requirement of at least 85% of the scheduled sessions for the intervention; (v) physically inactive, meaning they did not meet the international recommendations for moderate (<150 to 300 min) or vigorous (<75 to 150 min) physical activity [25]; and (vi) in physical condition compatible with the practice of physical activity. The exclusion criteria were (i) having any disability; (ii) suffering from a musculoskeletal injury or receiving physical rehabilitation therapy that prevented them from performing their usual physical activity; and (iii) being temporarily or permanently unable to perform physical activity.

### 2.4. Sample Size

Based on prior research [26,27], a clinically significant difference of 0.60 s in TUG, with a standard deviation of 0.20 s, was set as the threshold for the sample size calculation. The sample size calculation assumed an alpha level of 0.05, power of 85%, and a 20% attrition rate. This analysis suggested that 15 participants per group would be optimal.

### 2.5. Recruitment and Randomization

Participants were recruited from local health centers. After meeting the inclusion criteria, participants were randomly assigned to either the XKS group or the control group using the sealed-envelope lottery method. This process was managed via the Randomizer website (https://www.randomizer.org; accessed on 1 July 2023), ensuring random allocation.

### 2.6. Blinding

**Figure 1:**
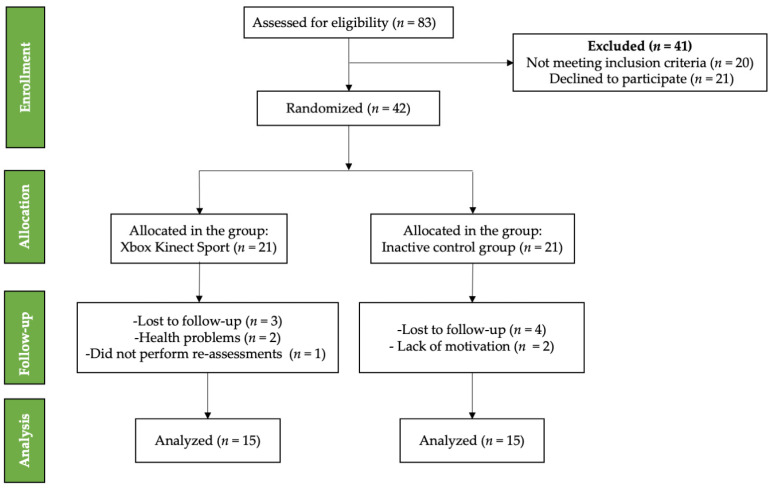
CONSORT flow diagram of participants included on the trial.

This study followed a single-blind design, with evaluators blinded to group allocation. Participants were aware of their group assignment, but the assessors who conducted the evaluations were unaware of whether participants were in the XKS group or the control group. Figure 1 summarizes the selection of participants.

### 2.7. Description of the Variables

Outcome measures encompassed the Mini-Mental State Examination (MMSE) to assess cognitive function, the Timed Up and Go (TUG), the Berg Balance Scale (BBS), and the Falls Efficacy Scale International (FES-I) for balance and fall risk evaluation. Additionally, the MIHS and MIPS (dominant and non-dominant hands) were employed to measure physical performance. Together, these assessments offered comprehensive insights into the participants’ cognitive, physical, and motor abilities.

### 2.8. Ethical Considerations

By signing the informed consent form, all participants acknowledged the inclusion criteria for the data’s usage and treatment. The protocol was created in accordance with the Declaration of Helsinki and approved by the scientific ethics committee of the Universidad Católica del Maule, Chile (Approval number: N°29-2022).

### 2.9. Anthropometric Parameters and Sociodemographic Assessments

Height was measured using a stadiometer (Seca model 220, SECA, Hamburg, Germany) with an accuracy of 0.1 cm, and weight was determined with a mechanical scale (Scale-Tronix, Chicago, IL, USA) accurate to 0.1 kg, with participants wearing minimal clothing [28]. The baseline characteristics of the sample are shown in Table 1.

### 2.10. Mini-Mental State Examination

The MMSE is a commonly used tool to assess the general cognitive status of individuals, t consists of a series of questions and tasks covering several cognitive areas, including temporal and spatial orientation, memory, attention, calculation, recall ability, and language, participants receive a total score, which ranges from 0 to 30 points, with a low score indicating possible cognitive impairments [29]. The test was administered by a trained and experienced evaluator, who performed a pre- and post-intervention measurement for each participant. Statistical analysis was based on the scores obtained in the MMSE, comparing the pre- and post-intervention differences.

### 2.11. Timed Up-and-Go Test

The TUG test was performed following established guidelines [30]. Participants were instructed to rise from a chair with arm support, walk across a 3 m path, turn, and return to the chair. The test was repeated three times, and the best time was recorded. Two assessors used single-beam photocells (Brower Timing System, Draper, UT, USA) to measure the time, and statistical analysis was based on the best of the three attempts.

### 2.12. Berg Balance Scale

The BBS was conducted following standard procedures as outlined [31]. This scale consists of 14 simple balance-related tasks, such as standing up from a chair, standing on one foot, and reaching forward. Each task is scored from 0 to 4, with 0 indicating the lowest level of performance and 4 indicating the highest. The total score ranges from 0 to 56, with higher scores indicating better balance. The assessment was performed by two trained assessors, and the final score was calculated as the sum of the individual task scores. Statistical analysis was conducted on the pre- and post-intervention scores to determine changes in balance performance.

### 2.13. Falls Efficacy Scale-International

The FES-I was administered following the established procedure [32]. The FES-I assesses the individual’s concern about falls during 16 different activities of daily living, such as walking around the house, bathing, and climbing stairs. Each activity is rated on a scale from 1 (not at all concerned) to 4 (very concerned). The test was administered by two trained raters who explained the purpose of the scale and ensured that each participant understood the items. Statistical analysis was performed pre- and post-intervention on each participant’s total scores.

### 2.14. Maximal Isometric Handgrip Strength

According to previous recommendations, the MIHS protocol was applied [33]. The optimal testing position involved sitting with the wrist and forearm in a neutral alignment, the elbow bent at a 90-degree angle close to the body, the spine straight, and the shoulder in a neutral position. The assessment utilized a portable dynamometer (Jamar^®^, PLUS+, Sammons Preston, Patterson Medical, Warrenville, IL, USA), set to the first position [34]. This configuration ensured contact between the first phalanx of the thumb and index finger, providing a secure grip on the device while maintaining proper alignment of the metacarpophalangeal and interphalangeal joints according to the size of the participant’s hand. Each participant performed three attempts per hand, with a 120-s rest period between trials.

### 2.15. Maximal Isometric Pinch Strength

Previous recommendations that MIPS was used [35]. The optimal testing position was found to be seated, with the wrist and forearm in a neutral position, the elbow flexed at a 90-degree angle, and the shoulder in neutral alignment. A portable pinch dynamometer (Jamar^®^ Hydraulic Pinch Gauge, Sammons Preston, Warrenville, IL, USA) was used for assessment. The device was positioned to allow contact between the distal phalanges of the thumb and index finger, allowing for a secure pinch while maintaining proper joint alignment of the metacarpophalangeal and interphalangeal joints. Participants performed 3 trials with each hand, with a 120-s rest between each attempt to minimize fatigue.

### 2.16. Intervention

The intervention protocols were based on the methodologies described in prior research [24,25]. The program was conducted at a community center for older people and facilitated by a trained physical education instructor. Participants underwent a one-week familiarization phase with the activities, consisting of three 60 min sessions, before being assigned to groups through randomization.

**Figure 2:**
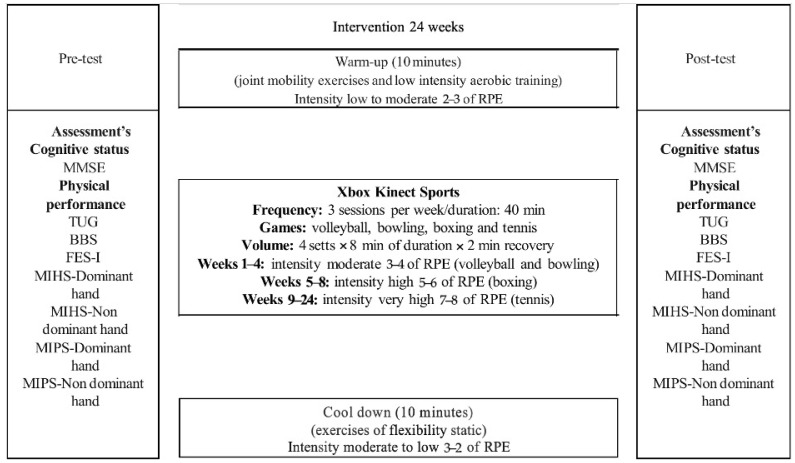
Assessments and regular sessions of the intervention. BBS: Berg Balance Scale; FES-I: Falls Efficacy Scale-International; MIHS: Maximal Isometric Handgrip Strength; MIPS: Maximal Isometric Pinch Strength; MMSE: Mini-Mental State Examination; RPE: Rating of Perceived Exertion; TUG: Timed Up-and-Go.

The XKS version 2 training sessions included exergames such as volleyball, bowling, boxing, and table tennis. Each game was performed for 8 min, followed by a 2 min rest period. To play, participants needed to position themselves within a 3.5 m motion range in front of a sensor placed below a television screen [36,37]. The training intensity was tracked using the 10-point Rating of Perceived Exertion scale [38]. During the initial 4 weeks, participants exercised at moderate intensity levels (3–4), progressing to higher intensities (5–6) in weeks 5 to 8 and reaching very high intensities (7–8) from weeks 9 to 24. Game complexity was gradually increased by advancing through levels, which demanded faster movements and greater effort, resulting in higher training intensities. Sessions were supervised by a physical education instructor to ensure safety and adherence to the protocol. Meanwhile, the CG engaged in their usual activities of daily living. Figure 2 provides a summary of the interventions.

### 2.17. Statistical Analysis

The data were analyzed using the statistical software SPSS version 25.0 (SPSS Inc., Chicago, IL, USA). Descriptive statistics, including mean values and standard deviations, were computed for all variables. The Shapiro-Wilk test was employed to verify the normality of the data distribution. To examine the interaction between time × group, a two-way mixed ANOVA with repeated measures was performed for the MMSE, TUG, BBS, FES-I, and MIHS for dominant and non-dominant hands, as well as the MIPS for both hands. When significant time × group interactions were found, post hoc comparisons using Bonferroni’s test were performed to examine intra-group (pre vs. post assessments) and inter-group (XKS group vs. CG) differences. The effect size for the time × group interaction was determined using partial eta squared (ηp^2^), with thresholds of 0.01, 0.06, and 0.14 indicating small, moderate, and large effects, respectively [36]. For post hoc comparisons, Cohen’s d was applied to assess effect sizes, with values of ≥0.2, ≥0.5, and ≥0.8 representing small, moderate, and large effects, respectively, and values greater than 1.0 indicating very large effects [39]. An alpha level of 0.05 was used for all statistical analyses.

## 3. Results

Table 2 presents the pre- and post-intervention results for the variables analyzed in the XKS group and CG. A two-way mixed ANOVA revealed a significant time × group interaction for the MMSE (F_(2,18)_ = 14.8; *p =* 0.000; ηp^2^ = 0.292, *large effect*), TUG (F_(2,18)_ = 19.5; *p =* 0.000; ηp^2^ = 0.351, *large effect*), and FES-I (F_(2,18)_ = 6.55; *p =* 0.015; ηp^2^ = 0.154, *large effect*). However, no significant interaction was found for the BBS (F_(2,18)_ = 0.998; *p =* 0.324; ηp^2^ = 0.027, *small effect*) or MIHS for both the dominant (F_(2,18)_ = 0.163; *p =* 0.688; ηp^2^ = 0.005, *small effect*) and non-dominant (F_(2,18)_ = 0.012; *p =* 0.912; ηp^2^ = 0.000, *small effect*) hands, besides in the MIPS for both the dominant (F_(2,18)_ = 0.099; *p =* 0.756; ηp^2^ = 0.003, *small effect*) and non-dominant (F_(2,18)_ = 0.233; *p =* 0.632; ηp^2^ = 0.006, *small effect*) hands.

**Figure 3:**
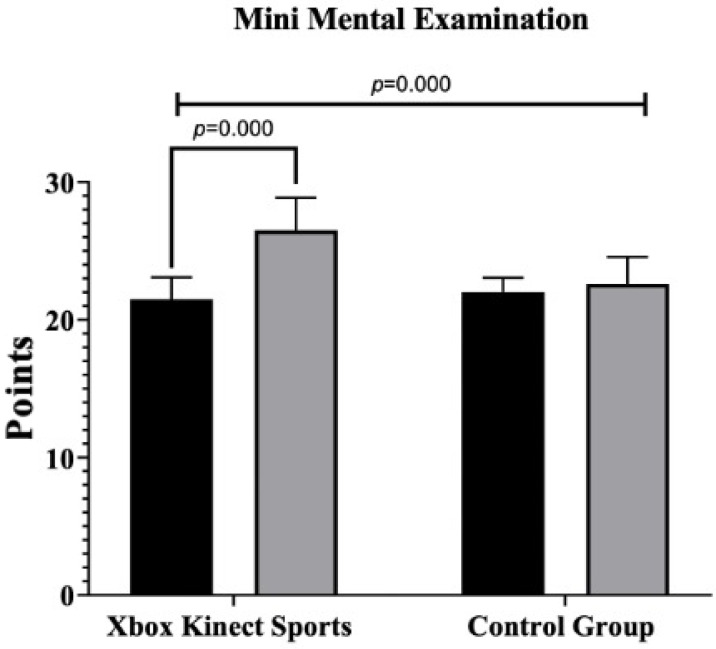
Multiple intragroup and intergroup comparisons of Xbox Kinect Sports and control group on cognitive status in physically inactive older females. MMSE: Mini-Mental State Examination.

**Figure 4:**
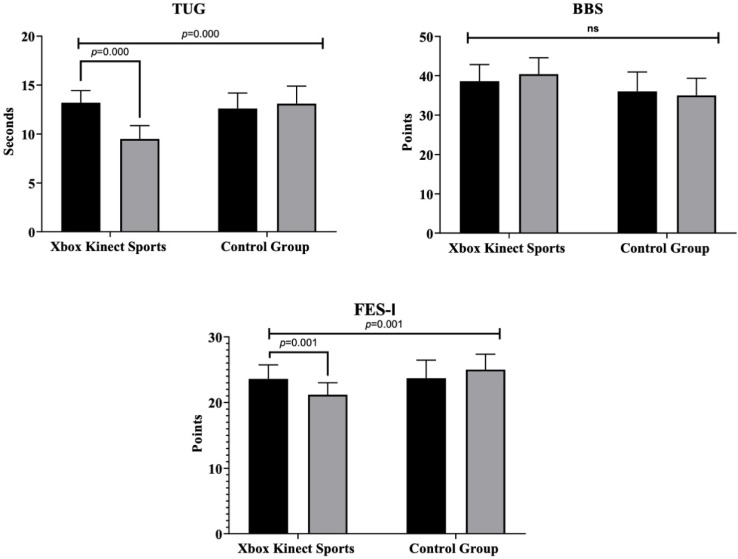
Multiple intragroup and intergroup comparisons of Xbox Kinect Sports and control group on physical performance physically inactive older females. BBS: Berg Balance Scale (*p* = 0.324); FES-I: Falls Efficacy Scale-International (*p* = 0.015); TUG: Timed Up-and-Go (*p* = 0.000). ns: no significant differences across the groups.

The results of the intragroup and intergroup comparisons are presented in Figure 3 and Figure 4. Regarding cognitive status measured by the MMSE, significant improvements were observed in the XKS group after the intervention (F = 14.8; *p =* 0.000; ηp^2^ = 0.292, *large effect*), with no significant changes in the CG (Alpha de Cronbach = 0.83). In TUG, a significant decrease in time was found in XKS group (F = 19.5; *p =* 0.000; ηp^2^ = 0.351, *large effect*), indicating improved physical performance, while CG did not show significant changes. For the FES-I, XKS group exhibited a significant reduction in scores (F = 6.550; *p =* 0.015; ηp^2^ = 0.154, *large effect*), reflecting reduced fear of falling, while CG no significant changes.

## 4. Discussion

This study aimed to compare the effects of XKS concerning inactive CG on cognitive status, as measured by the MMSE, and physical performance, as assessed by TUG, BBS, FES-I, MIHS and MIPS, in older females. The results showed significant improvements in favor of the XKS group versus the CG in the MMSE, and a significantly reduced time in the TUG, and fear of falling, as measured by FES-I. However, no significant changes were observed in the BBS, MIHS, or MIPS scores, either between or within groups. Therefore, our hypothesis is corroborated.

### 4.1. Mini-Mental State Examination

Regarding cognitive status, significant improvements in MMSE scores were observed in favor of the XKS group compared to the CG. The findings of the current study align with those reported by Htut et al. [40] in their RCT, where the experimental group using the XKS demonstrated significant cognitive improvements on the MMSE compared to the inactive CG (*p* < 0.001).

The intervention lasted 30 min per day, 3 non-consecutive days per week for 8 weeks in apparently healthy older people. The network meta-analysis for cognitive outcomes included 28 studies with cognitively healthy older people and 13 studies with patients with mild cognitive impairment. It provided data for four indirect comparisons, video game exercise vs. cognitive training, video game exercise vs. sequential training, sham intervention vs. passive control, and sequential vs. simultaneous training. All conditions, except for sham interventions, were significantly more effective than passive control in improving cognitive status, with *g*-values ranging from 0.18 to 0.43 [41]. Another study showed that interventions with XKS, based on adventure games, compared to inactive CG undergoing conventional physical therapy, were conducted twice a week for 60 min over a period of 7 weeks. The results indicated significant improvements in cognitive status (*p* < 0.05) in both groups, attributed to the inclusion of physical activity in both interventions [42]. Advances in neuroscience have demonstrated the importance of physical activity in our activities of daily living, as evidenced by a meta-analysis, Firth et al. [43] found that physical activity groups, compared to an inactive CG, showed significant effects on the hippocampus and cognitive status, a neural structure crucial for learning and overall cognitive function (*p* = 0.003). One of the most important neurological changes when combining physical and cognitive activity is the increase in brain-derived neurotrophic factor, which is crucial for neurogenesis and neuroplasticity throughout the lifespan and for preventing brain aging in older people [44]. Integrating exergaming as a therapeutic tool in professional interventions could enhance motivation and lead to significant improvements in cognitive functions.

### 4.2. Timed Up-and-Go Test

Significant improvements in TUG were reported in favor of XKS group compared to CG. In an RCT, similar results were found, with the TUG showing significant improvement in the Multicomponent Exercise Program group compared to the inactive CG (*p* < 0.001), and further notable progress at 12 and 24 weeks (*p <* 0.01), these findings demonstrate the program’s effectiveness in enhancing mobility in both healthy and frail older people [45]. The results obtained are like those reported by López-López et al. [46], who conducted a 12-week cognitive–motor training intervention in institutionalized older people. Their study observed significant improvements in TUG performance (*p* < 0.001) in the experimental group compared to an inactive CG. This is like the findings reported by Htut et al. [40], where the experimental group using the XKS showed a reduced time in the TUG compared to the inactive CG (*p* < 0.001). Our favorable results can be explained by the fact that motivating physical activities lead to changes in neurotransmitters such as dopamine, which plays a key role in motor control, motor regulation, and modulation of affective and emotional activities, impacting motor skills such as balance and walking speed [47]. Although favorable performance on the TUG test has traditionally been associated with the peripheral muscle capacity of the lower limbs in older people [48]. The current literature highlights that motor skills are a combination of motor control exerted by the central nervous system and its ability to coordinate peripheral muscle function, impacting balance and stability in standing and walking in older people [47]. This study emphasizes the positive impact of exergaming and multicomponent exercise programs on mobility, notably by improving balance and walking speed, as reflected in enhanced TUG performance among older people.

### 4.3. Berg Balance Scale

Regarding balance, no significant improvements in BBS were observed in either XKS group or CG. However, a meta-analysis by Chen et al. [49], where they included 12 RCTs with a total of 482 older people, the majority of studies implemented balance exercise-based interventions using exergames, participants who completed these interventions showed significant improvements in balance measured through the BBS (*p* < 0.001), compared to those who received usual care, the interventions lasted 8 to 24 weeks, with a frequency of 2 to 3 sessions per week. Similar results were obtained in an 8-week RCT, where three groups of healthy older people were studied by comparing voluntary stepping under stable and unstable conditions with a control group, and participants underwent game-based exercise training three times a week for 40 min per session. The results showed that reactive balance improved only in the unstable training group (*p* < 0.05), while functional balance improved in both the stable and unstable training groups (*p* < 0.05) [50]. A possible explanation for the lack of significant improvements in our study lies in the fact that XKS does not provide an unstable surface but instead maintains the same bipedal stability for the participants. This is further supported by the findings of Harris et al. [51] and Morat et al. [50], who reported improvements in postural balance, balance reactions, and righting responses in interventions utilizing unstable surfaces, as these required a higher level of activation and postural adjustment to perform the exergames in older people.

### 4.4. Falls Efficacy Scale-International

Regarding fear of falling, significant improvements in FES-I scores were observed in favor of XKS group compared to CG. Similar results were found in a study that implemented a 12-week interactive physical-cognitive training program, with 60 min sessions 3 times per week, significantly reduced the fall risk in older people (*p* = 0.015) compared to an inactive CG [52]. Similar findings were observed in another RCT, where the experimental group using the XKS exercise game showed a significant improvement in the FES-I score compared to inactive CG (*p* = 0.036), the intervention, aimed at apparently healthy older people [40]. A meta-analysis of 12 RCTs involving 482 older people found that exergame-based balance exercises significantly improved fall prevention (*p* = 0.009) compared to usual care. Most programs lasted 8 to 24 weeks with 2 to 3 sessions per week [49]. In addition, Lee et al. [53] conducted a meta-analysis on interactive virtual reality training for frail older people, finding significant reductions in fall risk (*p* < 0.001) and improvements in muscle strength, walking speed, and balance. The fall risk is related to cognitive and motor function, this entails maintaining adequate muscular and cardiovascular health. If these functions are enhanced, the risk of falling and dependence on activities of daily livings in older people would be reduced [54]. Functional exercise should be prioritized in interventions aimed at protecting older people at high fall risk, as well as enhancing and preserving their functional capacity [55]. This study underscores the effectiveness of exergaming and interactive physical-cognitive training in significantly reducing fear of falling and fall risk among older people.

### 4.5. Maximal Isometric Handgrip Strength and Maximal Isometric Pinch Strength

No significant improvements in MIHS and MIPS were observed for either XKS group or CG. This finding is consistent with Tuan et al. [56], who reported no changes in MIHS after a 12-week exergame program. However, Campo-Prieto et al. [57] found significant improvements in MIHS following a 10-week immersive virtual reality exergame program. Additionally, a meta-analysis by Ramsey et al. [58] demonstrated that higher physical activity levels were associated with better MIHS. These studies suggest that exergames involving moderate-to-vigorous physical activity may enhance MIHS in older people [57,58]. Regarding MIPS, no significant improvements were observed in either the XKS group or CG. Similarly, Keogh et al. [59] reported no significant differences in MIPS between experimental and control groups after a unilateral strength training program in older male. However, Ranganathan et al. [60], who specifically targeted MIPS in the dominant hand, observed significant improvements in motor control of the fingers, particularly in the medial-thumb pair, with notable reductions in force fluctuations (*p* < 0.05). The application of training involving moderate- to vigorous-intensity physical activity appears to have more beneficial effects, which could explain our results in MIHS and MIPS.

### 4.6. Limitations and Strengths

Our study has several limitations: (i) It included an inactive CG, which limits the ability to determine the relative effectiveness of the intervention compared to alternative active treatments; (ii) it did not account for participants’ dietary intake, which may have influenced the results; (iii) it did not measure physiological variables (e.g., heart rate, oxygen consumption), which could have affected the outcomes; and (iv) the study was conducted exclusively with females of a specific age group, limiting the generalizability of the findings to other populations. Despite these limitations, the study has several strengths: (i) It employed randomization of participants, enhancing the internal validity of the findings; (ii) it used valid instruments to assess cognitive function (MMSE) and physical performance (TUG, BBS, FES-I, MIHS, and MIPS), ensuring reliable and accurate measurement of outcomes; and (iii) it contributes to the growing body of evidence supporting the use of exergames as a beneficial health intervention for aging populations.

### 4.7. Practical Applications

XKS training is an effective and accessible approach to enhancing cognitive status and physical performance in activities of daily living among older people. The observed improvements performance in the MMSE, TUG, and FES-I demonstrate its potential to improve cognitive status, reduce fall risk, and foster greater independence. Its affordability and ease of implementation make XKS a practical choice for community centers and preventive health initiatives. Additionally, the wide variety of movements in its games makes XKS a cost-effective and engaging intervention for older people [13].

## 5. Conclusions

XKS significantly improves cognitive status, as measured by the MMSE, and physical performance through reduced time in TUG and fear of falling through the FES-I in physically inactive older females. Therefore, we recommend the use of active exercise games through XKS as an alternative to training for older people.

## Figures and Tables

**Table 1:** Baseline anthropometric parameters and sociodemographic assessments of physically inactive older females.

Variable	Assessment	XKS Group (n = 15)	CG (n = 15)
Age	Years	72.2 (6.76)	76.2 (2.03)
Anthropometric parameters	Bipedal height (m)	1.58 (0.07)	1.55 (0.06)
	Body mass (kg)	68.4 (29.4)	67.5 (4.69)
Academic level	Primary (%)	18	19
	Secondary (%)	15	10
	Bachelor (%)	4	3
	Postgraduate (%)	0	0
Civil status	Married (%)	30	12
	Separated (%)	6	4
	Widowed (%)	2	2
	Single (%)	0	0

CG: control group; XKS: Xbox Kinect sports. Data are presented in mean and standard deviation.

**Table 2:** Time × group interaction in the analyzed variables of Xbox Kinect Sport, and inactive control group on cognitive status and physical performance in physically inactive older females.

Assessment	Group	Pre		Post		Time × Group (F-Value)	Time × Group (*p*-Value)	ηp^2^	Effect Sizes
		Mean	SD	Mean	SD				
MMSE (points)	XKS	21.5	1.58	26.5	2.36	14.8	0.000	0.292	Large effect
	CG	22	1.05	22.6	1.95				
TUG (s)	XKS	13.2	1.22	9.5	1.35	19.5	0.000	0.351	Large effect
	CG	12.6	1.57	13.1	1.79				
BBS (points)	XKS	38.6	4.24	36	4.92	0.998	0.324	0.027	Small effect
	CG	40.4	4.16	35	4.34				
FES-I (points)	XKS	23.6	2.11	21.2	2.81	6.550	0.015	0.154	Large effect
	CG	23.6	2.75	25	2.35				
MIHS—Dominant hand (kg)	XKS	18.1	2.68	19	3.12	0.163	0.688	0.005	Small effect
	CG	15.6	1.50	15.9	1.66				
MIHS—Non dominant hand (kg)	XKS	16.6	3.23	16.7	3.0	0.012	0.912	0.000	Small effect
	CG	14.3	2.35	14.6	2.71				
MIPS—Dominant hand (kg)	XKS	7.70	1.88	7.40	1.54	0.099	0.756	0.003	Small effect
	CG	6.20	1.13	6.21	1.14				
MIPS—Non dominant hand (kg)	XKS	6.30	2.21	6.33	1.82	0.233	0.632	0.006	Small effect
	CG	5.1	2.02	5.7	2.76				

ηp^2^: Partial eta squared; BBS: Berg Balance Scale; CG: Control group; FES-I: Falls Efficacy Scale-International; MIHS: Maximal Isometric Handgrip Strength; MIPS: Maximal Isometric Pinch Strength; MMSE: Mini-Mental State Examination; SD: standard deviation; TUG: Timed Up-and-Go; XKS: Xbox Kinect sports.

## Data Availability

The data presented in this study are available on request from the corresponding author due to ethical restrictions.
